# Hepatotoxicity and Antimicrobial Resistance to Amoxicillin and Amoxicillin/Clavulanic Acid: Data Analysis from EudraVigilance

**DOI:** 10.3390/molecules30183825

**Published:** 2025-09-21

**Authors:** Ilaria Ammendolia, Carmen Mannucci, Emanuela Esposito, Gioacchino Calapai, Mariaconcetta Currò, Paola Midiri, Luigi Cardia, Fabrizio Calapai

**Affiliations:** 1 Department of Chemical, Biological, Pharmaceutical and Environmental Sciences, University of Messina, 98122 Messina, Italy; ilaria.ammendolia@unime.it (I.A.); emanuela.esposito@unime.it (E.E.); 2 Department of Clinical and Experimental Medicine, University of Messina, 98122 Messina, Italy; mariaconcetta.curro@unime.it (M.C.); paola.midiri@gmail.com (P.M.); 3 Department of Biomedical and Dental Sciences and Morphological and Functional Imaging, University of Messina, 98122 Messina, Italy; carmen.mannucci@unime.it (C.M.); fabrizio.calapai@unime.it (F.C.); 4 Department of Human Pathology of Adult and Childhood “Gaetano Barresi”, University of Messina, 98122 Messina, Italy; luigi.cardia@unime.it

**Keywords:** amoxicillin, amoxicillin/clavulanic acid, antimicrobial resistance, antibiotic resistance, hepatotoxicity, liver toxicity, pharmacovigilance, adverse reactions

## Abstract

Amoxicillin is widely prescribed, either as a monotherapy or in combination with clavulanic acid, with its therapeutic indications including a broad range of infections. Its combination with clavulanic acid maintains its known activity against strains producing β-lactamase. There are limited studies on adverse reactions and antibiotic resistance, with researchers relying primarily on databanks that report spontaneous events caused by amoxicillin or the combination of amoxicillin/clavulanic acid. Antimicrobial resistance is an overlooked adverse event, and pharmacovigilance databases could serve as a tool in tracking resistance. On this basis, a study to define the safety profile of amoxicillin and amoxicillin/clavulanic acid and to increase the knowledge necessary to support the battle against antimicrobial resistance was undertaken through the analysis of pharmacovigilance databases. Suspected adverse reactions to amoxicillin and to the combination of amoxicillin/clavulanic acid in the data system EudraVigilance (2020–2024) were analyzed. The most frequent alerts concerned “Skin and subcutaneous disorders” for both drugs. Disproportionate analysis of cases concerning “Hepatobiliary disorders” or “Drug inefficiency” indicates a significant increase in these alerts with the amoxicillin/clavulanic acid combination compared to amoxicillin. The amoxicillin/clavulanic acid combination has previously been associated with a higher risk of hepatotoxicity and antibiotic resistance to amoxicillin/clavulanic acid; however, this is the first time that a post-marketing surveillance study has shown that antimicrobial resistance is more likely to occur with the combination in comparison to amoxicillin alone.

## 1. Introduction

The semisynthetic combination of penicillin–amoxicillin was generated with the addition of an extra amino group to penicillin. It is a broad-spectrum antibiotic characterized by its core beta-lactam ring and a p-hydroxyphenylglycylamino side chain, giving it the molecular formula C_16_H_19_N_3_O_5_S. This structural modification was primarily aimed at improving absorption and spectrum and proved to be functional against the development of antibiotic resistance [1]. This drug, either alone or combined with the b-lactamase inhibitor clavulanic acid, is the most widely prescribed penicillin in Europe and many other countries [2]. Amoxicillin binds irreversibly to penicillin-binding protein 1A, which is fundamental for bacterial cell wall synthesis. This binding inactivates penicillin-binding protein 1A and leads to cell lysis [3]. Clavulanic acid is a β-lactam that binds irreversibly to the active site of many beta-lactamases, leading to enzyme inactivation. It is a β-lactam–oxazolidine compound with the molecular formula C_8_H_9_NO_5_, and its core structure consists of an azabicyclo [3.2.0]heptane ring system, featuring a β-lactam ring and an oxazolidine ring [4]. Therapeutic indications for amoxicillin include infections induced by beta-lactamase–negative bacteria, such as higher and lower respiratory tract infections, urinary tract infections, *Helicobacter pylori* treatment, and skin infections [5]. The combination of amoxicillin with clavulanic acid maintained amoxicillin’s known activity against β-lactamase-negative strains and reinstated its action against β-lactamase-producing strains. Furthermore, the combination expanded amoxicillin’s action against other strains producing β-lactamase, such as *Klebsiella pneumoniae* and anaerobic *Bacteroides fragilis* [6]. It has also been shown to be efficacious against urinary, respiratory, and soft tissue infections caused by β-lactamase-producing bacteria, as well as in gonorrhea and chancroid treatment [7]. Amoxicillin and amoxicillin/clavulanic acid are generally considered as well-tolerated medicinal products with an established safety profile, based on extensive clinical worldwide use. However, it is known that patients with recognized hypersensitivity to penicillin should not be treated with amoxicillin. Furthermore, the combination of amoxicillin/clavulanate should be prescribed with caution in patients presenting hepatic dysfunction, and it is not recommended in patients with a previous history of cholestatic jaundice/hepatic dysfunction [8]. The molecular and chemical aspects explaining the mechanism inducing hepatotoxicity and penicillin-associated multidrug resistance (beta-lactam antibiotic) have been previously described [9].

In the research literature, studies using real-world data and investigating adverse reactions and antibiotic resistance, based on databanks reporting spontaneous adverse reactions to medicinal products with amoxicillin and the combination of amoxicillin/clavulanic acid, are scarce [10]. It has also been suggested that antimicrobial resistance is an overlooked adverse event and that pharmacovigilance databases could serve as a tool in tracking antimicrobial use and resistance [11].

On this basis, a study defining the safety profile of adverse reaction signaling and investigating the potential role of pharmacovigilance databases in supporting the battle against antimicrobial resistance was undertaken. This study’s aim was to analyze spontaneous reports of suspected adverse reactions (SARs) to amoxicillin and amoxicillin/clavulanic acid in EudraVigilance (2020–2024), with a particular focus on hepatobiliary disorders and reported lack of efficacy, to explore both drug safety and potential antimicrobial resistance signals.

## 2. Results

### 2.1. Percentage and Ratio of Serious/Non-Serious Suspected Adverse Reactions

A total of 10,329 and 7900 Individual Case Safety reports (ICSRs) related to prescriptions of a combination of amoxicillin/clavulanic acid and amoxicillin, respectively, were identified in the EudraVigilance database as adverse reaction reports in the period from January 2020 to 31 December 2024. Of these reports, 5114 ICSRs linked to amoxicillin/clavulanic acid prescription were categorized as serious cases (a total of 49.5% of the total number of ICSRs were combination-related). Serious ICSRs related to amoxicillin comprised 3685 reports (a total of 46.6% of the total number of ICSRs were amoxicillin-related). An evaluation of the adverse reactions to the combination of amoxicillin–clavulanic acid and to amoxicillin shows that non-serious cases outnumbered serious cases for both drugs, with ratios of 0.98 (combination) and 0.87 (amoxicillin), respectively (Table 1).

### 2.2. Serious Suspected Adverse Reactions to Amoxicillin/Clavulanic Acid and Amoxicillin According to the System Organ Class (SOC) Level

**Figure 1:**
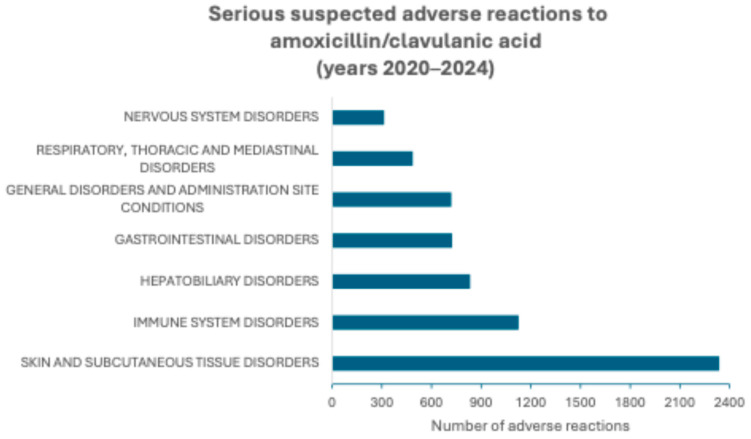
Serious suspected adverse reactions to the combination amoxicillin/clavulanic acid signaled in European Economic Area and United Kingdom in the years 2020–2024 and aggregated according to the System Organ Class (SOC) level.

**Figure 2:**
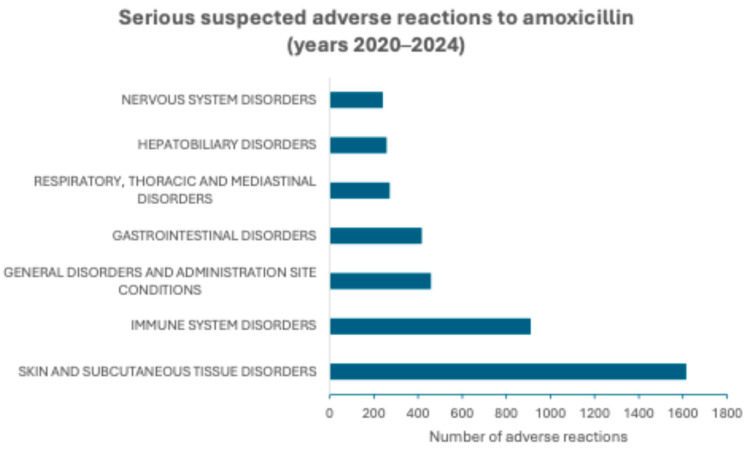
Serious suspected adverse reactions to amoxicillin signaled in European Economic Area and United Kingdom in the years 2020–2024 and aggregated according to the System Organ Class (SOC) level.

According to the SOC level, SAR aggregation to the amoxicillin/clavulanic acid combination and to amoxicillin shows that the most frequent adverse reactions among those signaled in the years 2020–2024 are grouped as “Skin and subcutaneous disorders” followed by “Immune system disorders” for both categories of medicinal products. However, after these two groups of SARs, the next most common group is “Hepatobiliary disorders” for the combination, while for amoxicillin, “General disorders and administration site conditions” is the third most common group of signaled SARs. These groups and the other groups of more frequently signaled SARs are represented in Figure 1 and Figure 2.

### 2.3. Sex Distribution of Cases Reporting Serious Suspected Adverse Reactions (SARs) to the Amoxicillin/Clavulanic Acid Combination and to Amoxicillin According to the System Organ Class (SOC) Level

Table 2 and Table 3 show the sex distribution of serious adverse reactions aggregated according to the SOC level based on the combination of amoxicillin/clavulanic acid and amoxicillin alone, respectively. Regarding the prescription of the combination drug, male patients reported more “Skin and subcutaneous tissue disorders” and “Hepatobiliary disorders”(Table 2), while for amoxicillin, female patients reported more “Skin and subcutaneous tissue disorders”, “Gastrointestinal disorders”, and “Respiratory, thoracic and mediastinal disorders” (Table 3). Disproportionate analysis of ICSRs concerning adverse reaction signaling to the combination of amoxicillin/clavulanic acid vs. the adverse reactions to amoxicillin, aggregated according to the SOC level and performed using the Reporting Odds Ratio (ROR), indicates an increase in the risk of “Hepatobiliary disorders” due to the use of the amoxicillin/clavulanic acid combination in comparison to that of amoxicillin alone (Table 4).

### 2.4. Evaluation of Adverse Reactions to Amoxicillin/Clavulanic Acid and Amoxicillin Categorized as “Hepatobiliary Disorders”

Serious reactions belonging to the SOC group “Hepatobiliary disorders” were detected in 257 and 834 cases for amoxicillin and the amoxicillin/clavulanic acid combination, respectively. Statistical analysis shows an asymmetric sex distribution, since “Hepatobiliary disorders” are more frequent in females treated with amoxicillin and in males treated with the amoxicillin/clavulanic acid combination (Table 5). Disproportionate analysis of serious ICSRs concerning “Hepatobiliary disorders” reports, which was performed using the Reporting Odds Ratio (ROR), indicates a significant increase in the risk of this adverse effect with the use of the combination of amoxicillin/clavulanic acid in comparison to that of amoxicillin alone (Table 6).

### 2.5. Evaluation of Adverse Reactions to Amoxicillin and Amoxicillin/Clavulanic Acid Categorized as “Drug Ineffective”

For this evaluation, the difference between serious and not serious cases was not maintained. The reaction categorized as “drug ineffective” was detected in 45 and 156 serious and non-serious cases for amoxicillin and for the amoxicillin/clavulanic acid combination, respectively. Statistical analysis shows that the frequencies of male and female cases are overlapping for amoxicillin, while the sex distribution between the two sexes for the combination of amoxicillin/clavulanic acid indicates that inefficiency against infections is more frequent in females (Table 7). Disproportionate analysis of ICSRs reporting the adverse reaction “Drug ineffective”, performed using the Reporting Odds Ratio (ROR), indicates an increase in the risk of this adverse effect with the use of the amoxicillin/clavulanic acid combination in comparison to that of amoxicillin alone (Table 8).

## 3. Discussion

In the present study, we analyzed data on SAR signaling related to amoxicillin and the combination of amoxicillin/clavulanic acid in the years 2020–2024 in European countries and the UK. These medicinal products show a similar serious/non-serious score of close to one, which is generally considered as acceptable [12]. “Serious” adverse events are defined as any untoward medical occurrence that, at any dose, results in death; requires hospital admission or prolongation of existing hospital stay; results in persistent or significant disability/incapacity; is life threatening; or results in cancer, congenital anomalies, or birth defects; or that would be regarded as serious if they had not responded to acute treatment [13].

The most frequent alerts in the EudraVigilance database, in the years taken into consideration, concern “Skin and subcutaneous disorders” followed by “Immune system disorders” for both the combination and amoxicillin alone. This is not a novel discovery, since a 10-month prospective cohort study that included all hospitalized patients and was designed to identify those with adverse cutaneous drug reactions showed that medicinal products containing amoxicillin had the highest reaction rate [14].

Other authors, through a mixed prospective–retrospective cohort study, found that the skin and subcutaneous system, together with the gastrointestinal system, was commonly affected by amoxicillin alone or in combination, while respiratory thoracic disorders, the nervous system, and general disorders were not usually involved [15]. The prevalence of “Skin disorders and subcutaneous disorders” (at least as far as pediatric age is concerned), limited to the combination of amoxicillin/clavulanic acid, can be explained by the presence of sodium benzoate, which is found in the suspension formulation as a preservative. According to this hypothesis, sodium benzoate probably acts through a non-immunologic mechanism, and care should be given to children allergic to sodium benzoate-containing pharmaceutical formulations [16].

Skin and immune disorders caused by medicinal products containing amoxicillin are probably closely related. Among all beta-lactams, amoxicillin is a frequently used drug for sensitization. Cutaneous amoxicillin-mediated reactions can be classified as immediate or delayed reactions; the former are believed to be mediated by IgE [17].

Among its various mechanisms, amoxicillin can alter immune system activity, and it has been shown that amoxicillin decreases phagocytosis and macrophage chemotaxis [18] and that early-life amoxicillin exposure can alter the immune response locally and systemically long after withdrawal [19].

The sex distribution shows the prevalence of female cases of “Gastrointestinal disorders” and “Respiratory, thoracic and mediastinal disorders” in SARs signaling to both amoxicillin/clavulanic acid and amoxicillin. Alerts for “Skin and subcutaneous tissue disorders” show an asymmetry in sex distribution for the two medicinal product categories, as there is a prevalence of female cases for amoxicillin alone compared to male cases for the combination. Disproportionate analysis performed using ROR indicates a potential increase in the risk of “Hepatobiliary disorders” due to the use of the amoxicillin/clavulanic acid combination in comparison to that of amoxicillin alone.

In the Medical Dictionary for Regulatory Activities (MedDRA), “Hepatobiliary disorders” represent a group of adverse reactions classified according to the System Organ Class (SOC). This group includes terms describing pathological conditions of the liver (hepato-) and of the biliary system (biliary). “Hepatobiliary disorders” are non-oncologic or oncologic disorders that affect the liver, bile ducts, and/or gallbladder. Examples include hepatitis, cirrhosis, and cholangitis [20].

Hepatobiliary adverse drug reactions are an important issue in the field of drug safety and pharmacovigilance, as they are the leading cause of acute liver failure in the United States and Europe [21,22]. Amoxicillin, either alone or in combination with clavulanic acid, has previously been described as a hepatotoxic substance in pediatrics [23]. Through two previous population-based studies in adults with drug-induced liver injury, other authors indicated that the combination of amoxicillin/clavulanic acid displays a higher risk of acute liver injury with respect to amoxicillin alone [24,25].

Amoxicillin-induced liver toxicity is not a direct pharmacological effect but primarily immunoallergic. This immune-mediated hypersensitivity leads to injury that can be more commonly cholestatic or less frequently hepatocellular. It has been suggested that Class I and Class II HLA genotypes affect susceptibility to amoxicillin/clavulanic acid-induced hepatic injury, indicating the importance of the adaptive immune response in the pathogenetic mechanism [26]. Recently, a retrospective study analyzing the ICSRs of the global pharmacovigilance database VigiBase, signaled in Switzerland from 2010 to 2020, indicated that the combination of amoxicillin/clavulanic acid was among the most frequently suspected drugs for severe drug-related hepatic disorders. The authors concluded that the cause of hepatotoxicity-induced by the prescription of the amoxicillin/clavulanate combination is still unknown, postulating an immunoallergic mechanism [27]. Analysis using EudraVigilance’s data seem to confirm that the combination of amoxicillin/clavulanic acid is less safe than amoxicillin alone, since the comparison of alerts for “Hepatobiliary disorders” against the combination showed, through an ROR of 2.78 (95% C.I. 2.41–3.24), a stronger association between liver disorders and amoxicillin/clavulanic acid-based medicinal product use.

It has been suggested that pharmacovigilance databases could serve as a tool in measuring antibiotic resistance, with the aim of tracking resistant microorganisms, reducing therapy failures, and, finally, ensuring appropriate prescription of existing antibiotics [28,29]. On this basis, part of the present research was devoted to identifying cases of antimicrobial resistance to the combination of amoxicillin/clavulanic acid and to amoxicillin alone in the database EudraVigilance. This research was conducted by identifying cases reporting the adverse reaction “Drug ineffective” for both the medicinal products in the years 2020–2024. According to MedDRA, the term “Drug ineffective” describes the situation where a drug fails to induce the expected therapeutic effect [30].

The combination of amoxicillin/clavulanic acid is prescribed more frequently than amoxicillin in many countries. Amoxicillin alone has fewer side effects and can be prescribed at higher oral doses [2]. Antimicrobial resistance occurs when the antibiotic is unable to treat certain bacterial infections because the pathogens causing these infections have developed mechanisms that prevent the drug from functioning, counteracting the antibiotic’s effectiveness. Mechanisms involved in resistance to amoxicillin treatment are target alteration, antibiotic inactivation, and reduced permeability to the drug [31]. This association offers broader coverage by combining amoxicillin, a penicillin derivative effective against Gram-positive and Gram-negative bacteria, with clavulanic acid, which counteracts β-lactamase-producing strains [32]. However, resistance to amoxicillin/clavulanic acid and amoxicillin remains a concern. Resistance is often linked to beta-lactamase production, but other mechanisms such as outer membrane protein modifications can play a role [33,34]. Disproportionate analysis of the association of the alert/adverse reaction “Drug ineffective” in the present work indicates a higher potential risk of inefficiency with the amoxicillin/clavulanic acid combination, with the ROR being 2.78 (95% C.I.: 1.92–3.73). The ROR is a disproportionality measure used to identify the association of an adverse event with a certain medicinal product upon exposure. When the ROR is more than one, this means that there is a greater probability that the adverse event occurred due to exposure to the medicinal product [35]. In our case, the results of the disproportionality analysis suggest that drug inefficiency is about 2.5 times more likely to occur with the amoxicillin/clavulanic acid combination in comparison with the use of amoxicillin alone. Since the efficiency of these two medicinal products is commonly measured against infections, this means that antibiotic resistance occurrence is probably more common with the combination.

This can be a problem because the use of amoxicillin and clavulanic in combination is considered to be significantly more effective than that of amoxicillin alone, and therefore, this combination is strongly recommended and regularly prescribed in clinical practice. Based on the evidence of numerous adverse reactions characterized by drug ineffectiveness and considering that not all cases are directly related to resistance, further investigations of this issue are necessary. Investigating potential differences in susceptibility between infectious processes and identifying factors that increase resistance could contribute to improving the rational use of this medicinal product.

The results produced with the present study need to be interpreted with care due to the known limitations of pharmacovigilance research using data systems of spontaneous alerts for drugs with known adverse reactions. Limitations include the arbitrary choice of the years analyzed, the lack of a denominator, under-reporting, lower-quality information, causal relationship uncertainty, and, finally, the difficulty in controlling confounding factors such as comorbidities or, sometimes, the dosage and frequency duration of exposure, which may have an influence on health/pathology conditions. Moreover, although care and attention were taken to identify and remove duplicates, they may still exist after data extraction.

A different safety profile for the two medicinal products has been previously described. Amoxicillin/clavulanic acid has been associated with a higher risk of Stevens–Johnson syndrome and purpura, as well as with a 9-fold higher average reporting rate for hepatitis than that for amoxicillin alone [10]. However, this is the first time that a post-marketing surveillance study has used real-world data of spontaneous reports on adverse reactions to show that antimicrobial resistance has a greater probability of occurring with the combination in comparison to amoxicillin alone.

In conclusion, beyond the limitations described above, this research focuses on two aspects related to the safety of prescribing amoxicillin and the amoxicillin/clavulanic acid combination. The data confirm previous research indicating that prescribing the amoxicillin/clavulanic acid combination carries an increased risk of hepatobiliary disorders. Moreover, the combination developed to combat amoxicillin resistance appears to be, in many cases, even less effective than amoxicillin itself in combating antibiotic resistance. This observation needs to be handled with caution because the preferred term “drug ineffective” shows indirect proof of antimicrobial resistance. Moreover, the combination amoxicillin-clavulanic acid is often administered as empirical treatment for hospitalized community-acquired pneumonia, while amoxicillin is often chosen for less severe infectious conditions treated at outpatient services [2,36]. In addition, potential alternative explanations of drug ineffectiveness include inappropriate dosing, non-bacterial infections, poor patient adherence, infections with intrinsically resistant organisms, and disease severity. These aspects must be considered to avoid overstating the association between this event and drugs. In summary, pharmacovigilance databases such as EudraVigilance could represent a useful tool for the study of antimicrobial resistance, but they cannot substitute the reports updated by microbiology centers.

However, despite the limitations outlined above and given the exploratory nature of the disproportionality analysis conducted in this study, which does not allow for a precise quantification of the identified risk, both aspects relating to the safety of amoxicillin/clavulanic acid use and hepatotoxicity on antibiotic resistance deserve further investigation.

## 4. Materials and Methods

EudraVigilance is a database containing suspected adverse reactions (SARs) related to medicines authorized for the European Union (EU) market. SARs are traceable in individual cases (Individual Case Safety Reports; ICSRs) and are signaled by national drug regulatory authorities in the EU or by marketing authorization holders. EudraVigilance collects reports of “suspected” adverse reactions, meaning unwanted medical events observed following medicine use that are not necessarily related to or caused by the medicine itself [37].

### 4.1. Design of the Study

In the present study, ICSRs reporting SARs that occurred in patients to whom amoxicillin or the combination amoxicillin/clavulanic acid was prescribed, collected from 1 January 2020 to 31 December 2024, were collected and analyzed. The public version of the EudraVigilance database was used, and data collection on SARs was conducted according to the following inclusion criteria: serious SARs and reports sent only from healthcare professionals in cases regarding all ages (from 0 to > 85 years) and those reported from the European Economic Area, including the United Kingdom. To evaluate the adverse reaction identified as “Drug ineffective”, all serious and non-serious cases to amoxicillin and to the combination were analyzed. We mention the UK separately because in EudraVigilance, it continues to be included within the EEA. Alerts were excluded from the analysis when they were reported by non-healthcare professionals or came from non-European countries. For all cases, information on patient characteristics (age group and sex), adverse reaction type (often more than one for each ICSR), and the qualification of the primary source was provided. Regarding the criteria for data extraction from ICSRs, SAR selection was based on the Medical Dictionary for Regulatory Activities (MedDRA) [38]. It is used to code cases of adverse effects in pharmacovigilance databases and to facilitate searches in databases on adverse drug reactions. Every mentioned SAR was extracted and counted for every single case. Adverse reactions were grouped under the terms of the SOC (System Organ Classification) level in the MedDRA hierarchy, such as musculoskeletal and connective tissue disorders or vascular disorders. The SOC system’s organ classification is the highest level in the hierarchy, as it captures the broadest concept useful for data retrieval. It is a way of grouping medical terms based on body systems or functions. The term “Drug ineffective”, used in this study has been used as a so-called “Preferred term” (PT) listed in MedDRA and reported by the National Center for Biomedical Ontology. A PT is a distinct descriptor (single medical concept) for an adverse symptom or sign. We selected all single adverse reactions recorded in the ICSRs as “Drug ineffective”, and we counted them all and analyzed their frequency for the amoxicillin/clavulanic acid combination and for amoxicillin alone.

### 4.2. Data Analysis

The source of data extraction is a line listing a structured table where each row represents an ICSR and each column represents a specific data point associated with that case. The data were analyzed by aggregating the PTs of individual reports to a higher level of the MedDRA hierarchy by merging individual serious SARs in the SOC level (e.g., nausea and vomiting are classified in the same group as “Gastrointestinal symptoms”). Only reports classified as serious were analyzed, except for the analysis of data related to the adverse reaction “Drug ineffective”. In accordance with the E2D guidelines of the International Council for Harmonization, ICSRs are classified as serious if they are life-threatening, have resulted in death, have resulted or prolonged hospitalization or disability, or are related to a congenital anomaly/birth defect or other medically important conditions. The adequate stratification of alerts by sex group was performed to avoid biases caused by confounding effects and to analyze these two variables separately. The sex distribution was analyzed using the chi-square test. Duplicate and incomplete ICSRs were excluded from the analysis. A duplicate search was conducted of the dataset based on similarity in terms of the adverse reaction, age, sex, suspected/interacting medicinal products, and the EudraVigilance local report number. The statistical analyses used were one-way ANOVAs. A disproportionate analysis of the potential association of the SOC group “Hepatobiliary disorders” and of the alert “Drug ineffective” was performed by the reporting odds ratio (ROR) and comparing the SARs of the amoxicillin/clavulanic acid combination with those signaled for amoxicillin alone. Disproportionality analysis was used to compare the proportions or frequencies of two or more groups and to verify whether the differences are statistically significant. It is a methodology used to detect adverse drug reaction alerts. It is based on the two-by-two contingency table. In this way, the differences between the occurrence frequency and the background frequency for target drugs and target adverse events can be compared [39]. The ROR calculates the odds ratio of a selected drug versus other drugs for a certain adverse event [40]. It is used here to establish the strength of disproportionality by comparing SARs signaled for the combination of amoxicillin/clavulanic with those signaled for amoxicillin alone. An ROR equal to 1 indicates the absence of an alert; conversely, an ROR greater than 1 indicates an alert and the existence of an association [41]. All statistical analyses were completed using the SPSS statistical software, version 29.0 (SPSS, IBM, Armonk, NY, USA).

## Figures and Tables

**Table 1:** Serious and non-serious Individual Cases Safety Reports related to the prescription of the combination amoxicillin/clavulanic acid and amoxicillin, signaled in the years 2020–2024 in the European Economic area and United Kingdom.

Medicinal Product	Total Number of ICSRs	Serious ICSRs	Non Serious ICSRs	Serious/Non Serious Ratio
Amoxicillin/ clavulanic acid	10,329	5114	5215	0.98
Amoxicillin	7900	3685	4215	0.87

**Table 2:** Sex distribution of serious suspected adverse reactions (SARs) related to the prescription of the combination amoxicillin/clavulanic acid signaled in the years 2020–2024 in the European Economic area and United Kingdom and aggregated according to the System Organ Class (SOC) level.

SOC	Males (N = 2238)	Females (N = 2579)	Male and Female Cases	% of All Serious Cases	Significance Level (*p*)
Skin and subcutaneous tissue disorders	1189 (50.8%)	1149 (49.2%)	2338	48.5%	0.0005 *
Immune system disorders	547 (48.5%)	580 (51.5%)	1127	23.4%	0.2087
Hepatobiliary disorders	453 (54.3%)	381 (45.7%)	834	17.3%	0.0001 *
Gastrointestinal disorders	300 (41.4%)	425 (58.6%)	725	15.0%	0.0107 *
General disorders and administration site conditions	322 (44.8%)	397 (55.2%)	719	14.9%	0.4007
Respiratory, thoracic and mediastinal disorders	202 (41.6%)	284 (58.4%)	486	10.1%	0.0395 *
Nervous system disorders	147 (46.8%)	167 (53.2%)	314	6.5%	0.9028

* = *p* < 0.05 vs. males.

**Table 3:** Sex distribution of serious suspected adverse reactions (SARs) related to the prescription of amoxicillin signaled in the years 2020–2024 in the European Economic area and United Kingdom and aggregated according to the System Organ Class (SOC) level.

SOC	Male Cases (N = 1658)	Female Cases (N = 2032)	Male and Female Cases	% of All Serious Cases	Significance Level (*p*)
Skin and subcutaneous tissue disorders	655 (40.6%)	959 (59.4%)	1614	43.7%	0.0034 *
Immune system disorders	383 (42.1%)	527 (57.9%)	910	24.7%	0.1226
General disorders and administration site conditions	186 (40.5%)	273 (59.5%)	459	12.4%	0.0737
Gastrointestinal disorders	161 (38.7%)	255 (61.3%)	416	11.3%	0.0158 *
Respiratory, thoracic and mediastinal disorders	98 (36.2%)	173 (63.8%)	271	7.3%	0.0054 *
Hepatobiliary disorders	124 (48.2%)	133 (51.8%)	257	7.0%	0.3017
Nervous system disorders	99 (41.2%)	141 (58.8%)	240	6.5%	0.2669

* = *p* < 0.05 vs. males.

**Table 4:** Reporting odds ratio (ROR) of Individual Cases Safety Reports (ICSRs) signaling serious adverse reactions to the combination amoxicillin/clavulanic acid vs. the adverse reactions to amoxicillin in European Economic Area and United Kingdom in the years 2020–2024. Suspected adverse reactions (SARs) are aggregated according to the System Organ Class (SOC) level.

SOC	Cases of SARs to Amoxicillin/Clavulanic Acid	All Other Cases of SARs to Amoxicillin/ Clavulanic Acid	Cases of SARs to Amoxicillin	All Other Cases of SARs to Amoxicillin	ROR of Cases of SARs to Amoxicillin/Clavulanic Acid vs. Amoxicillin (95% C.I.)
Skin and subcutaneous tissue disorders	2338 (22.63%)	7991	1614 (20.43%)	6286	1.13 (1.06–1.22)
Immune system disorders	1127 (10.91%)	9202	910 (11.52%)	6990	0.94 (0.86–1.03)
Hepatobiliary disorders	834 (8.07%)	9495	257 (3.25%)	7643	2.61 (2.26–3.01)
Gastrointestinal disorders	725 (7.02%)	9604	416 (5.26%)	7484	1.36 (1.20–1.54)
General disorders and administration site conditions	719 (6.96%)	9610	459 (5.81%)	7441	1.21 (1.07–1.37)
Respiratory, thoracic and mediastinal disorders	486 (4.70%)	9843	271 (3.43%)	7629	1.39 (1.19–1.62)
Nervous system disorders	314 (3.04%)	10,015	240 (3.04%)	7660	1.00 (0.84–1.19)

**Table 5:** Sex distribution of cases reporting “Hepatobiliary disorders” related to the prescription of the combination amoxicillin/clavulanic acid and amoxicillin, signaled in the European Economic Area and United Kingdom in the years 2020–2024.

Medicinal Product	Total Number of Serious Cases	Male Cases	Female Cases	Cases Reporting “Hepatobiliary Disorders”	Male Cases Reporting “Hepatobiliary Disorders”	Female Cases Reporting “Hepatobiliary Disorders”	Significance Level *p*
Amoxicillin/clavulanic acid	10,329	4558	5771	834	453	381	0.00001
Amoxicillin	7900	3219	4681	257	124	133	0.02211

**Table 6:** Reporting odds ratio (ROR) of Individual Cases Safety Reports (ICSRs) signaling “Hepatobiliary disorders” as adverse reaction to the combination amoxicillin/clavulanic acid vs. amoxicillin in European Economic Area and United Kingdom in the years 2020–2024.

Medicinal Product	Serious Cases of “Hepatobiliary Disorders”	All Other Serious Cases	ROR of Serious Cases of “Hepatobiliary Disorders” to Amoxicillin/Clavulanic Acid vs. Amoxicillin (95% C.I.)
Amoxicillin/clavulanic acid	834	3983	2.78 (2.41–3.24)
Amoxicillin	45	3433	

**Table 7:** Sex distribution of cases reporting the inefficiency of amoxicillin or of the combination amoxicillin/clavulanic acid, signaled in the European Economic Area and United Kingdom in the years 2020–2024. N.S. = not significant.

Medicinal Product	Total Number of Cases (Serious and Non Serious)	Male Serious and Non Serious Cases	Female Serious and Non Serious Cases	Cases Reporting “Drug Ineffective” as Adverse Reaction	Male Cases Reporting “Drug Ineffective” as Adverse Reaction	Female Cases Reporting “Drug Ineffective” as Adverse Reaction	Significance Level *p*
Amoxicillin/clavulanic acid	10,329	4558	5771	156	80	76	0.00001
Amoxicillin	7900	3219	4681	45	16	29	N.S.

**Table 8:** Reporting odds ratio (ROR) of Individual Cases Safety Reports (ICSRs) signaling “Drug ineffective” as adverse reaction to the combination amoxicillin/clavulanic acid vs. amoxicillin in European Economic Area and United Kingdom in the years 2020–2024.

Medicinal Product	Cases of “Drug Ineffective”	All Other Cases	ROR of Cases of “Drug Ineffective” to Amoxicillin/Clavulanic Acid vs. Amoxicillin (95% C.I.)
Amoxicillin/clavulanic acid	156 (1.51%)	10,173	2.68 (1.92–3.73)
Amoxicillin	45 (0.57%)	7855	

## Data Availability

The data analyzed and presented in this study are available on the public EudraVigilance data system. https://www.ema.europa.eu/en/human-regulatory-overview/research-development/pharmacovigilance-research-development/eudravigilance (accessed on 18 September 2025).

## Abbreviations

The following abbreviations are used in this manuscript:

SAR	Suspected adverse reaction
ICSR	Individual Case Safety Reports
SOC	System Organ Class
EEA	European Economic Area
ROR	Reporting Odds Ratio
HLA	Human leukocyte antigen
C.I.	Confidence intervals
EU	European Union
MedDRA	Medical Dictionary for Regulatory Activities
PT	Preferred Term
